# Multidrug-Resistant Hypervirulent *Klebsiella pneumoniae* Found Persisting Silently in Infant Gut Microbiota

**DOI:** 10.1155/2020/4054393

**Published:** 2020-10-24

**Authors:** I. Y. Vasilyev, I. V. Nikolaeva, M. N. Siniagina, A. M. Kharchenko, G. S. Shaikhieva

**Affiliations:** ^1^Institute of Fundamental Medicine and Biology, Federal University, 420008 Ulitsa Kremlyovskaya 18, Kazan, Russia; ^2^Kazan State Medical University, 420012 Ulitsa Butlerova 49, Kazan, Russia

## Abstract

Since the spread of multidrug-resistant *Klebsiella pneumoniae* (MDRKP) strains is considered as a challenge for patients with weakened or suppressed immunity, the emergence of isolates carrying determinants of hypervirulent phenotypes in addition may become a serious problem even for healthy individuals. The aim of this study is an investigation of the nonoutbreak *K. pneumoniae* emergence occurred in early 2017 at a maternity hospital of Kazan, Russia. Ten bacterial isolates demonstrating multiple drug resistance phenotypes were collected from eight healthy full-term breastfed neonates, observed at the maternity hospital of Kazan, Russia. All the infants and their mothers were dismissed without symptoms or complaints, in a satisfactory condition. Whole-genome shotgun (WGS) sequencing was performed with the purpose to track down a possible spread source(s) and obtain detailed information about resistance determinants and pathogenic potential of the collected isolates. Microdilution tests have confirmed production of extended-spectrum *β*-lactamases (ESBL) and their resistance to aminoglycoside, *β*-lactam, fluoroquinolone, sulfonamide, nitrofurantoin, trimethoprim, and fosfomycin antibiotics and *Klebsiella* phage. The WGS analysis has revealed the genes that are resistant to aminoglycosides, fluoroquinolones, macrolides, sulfonamides, chloramphenicols, tetracyclines, and trimethoprim and ESBL determinants. The pangenome analysis had split the isolates into two phylogenetic clades. The first group, a more heterogeneous clade, was represented by 5 isolates with 4 different *in silico* multilocus sequence types (MLSTs). The second group contained 5 isolates from infants born vaginally with the single MLST ST23, positive for genes corresponding to hypervirulent phenotypes: yersiniabactin, aerobactin, salmochelin, colibactin, hypermucoid determinants, and specific alleles of K- and O-antigens. The source of the MDRKP spread was not defined. Infected infants have shown no developed disease symptoms.

## 1. Introduction

Newborn babies could obtain microorganisms from clinical environment, personnel, other patients, and parents, e.g., via breast milk. Since *Enterobacteriaceae* are known as early human gastrointestinal tract colonizers, local and systemic diseases could take place during the dynamic microbiome development. Their stabilization and persistence may have destructive consequences on the host's vital functions. Moreover, due to high horizontal gene transport frequency between the microbiome members, persistence of even a single strain carrying pathobiotic genes after its spread may cause explicit cytotoxic and genotoxic effects on host cells, leading to dangerous repercussion including colorectal cancer in particular [1]. Underweight and weakened patients of neonatal intensive care nurseries often suffer from Gram-positive bacterial infections, mostly caused by coagulase-negative *Staphylococci*, while the infections caused by Gram-negative bacteria are considered as more rare and deadly even after more rapid generalization [2].

*Klebsiella pneumoniae* is a causative agent of numerous nosocomial and community acquired infections including pneumonia, sepsis, bacteremia, meningitis, pyogenic liver abscesses, urinary tract infections, and more. The risk group historically includes patients with a weakened and malfunctioning immune system, but the spread of hypervirulent strains also endangers immunosufficient individuals [3]. First isolated from the lungs of patients with pneumonia *postmortem*, *K. pneumoniae* were acknowledged as a part of the normal human gastrointestinal tract microbiome since then. The colonization can spread, persist for years, and cause different pathologies from hidden carriage to fatal acute infections even for healthy individuals [4].

In this study, we applied whole-genome shotgun (WGS) sequencing to describe in detail 8 cases of asymptomatic carriage of multidrug-resistant *K. pneumoniae* (MDRKP) in the gastrointestinal tract of full-term infants, detected at similar time periods after the birth without visible symptoms in their health.

## 2. Materials and Methods

### 2.1. Ethical Approval

The infant stool samples collection was performed as a routine task during normal observation at the maternity hospital. The standard biosecurity and institutional safety procedures have been adhered to handle human subjects. Informed consent was obtained from all individual participants' parents included in the study. No special ethical restrictions were raised.

### 2.2. Isolation of MDRKP from the Infants' Stool Samples

#### 2.2.1. Patients

A total of 10 *K. pneumoniae* isolates demonstrating a multidrug resistance phenotype were collected from 10 stool samples (8 nonrepetitive) from 8 newborn full-term breastfed infants during hospitalization in a maternity hospital of Kazan, Russia. 5 neonates were born with vaginal delivery, 3 after cesarean surgery. No infection outbreak was recorded, and no detailed microbiological analysis was performed on parents. The infant stool samples were collected at 3–4 days of life and were contaminated with 10^8^–10^9^ of MDRKP colony-forming units per gram of stool. The meconium samples obtained from all 8 individuals were not tested for contamination. Yet, the meconium samples obtained from 40 infants in the previous research studies were conditionally sterile or contained bifidobacteria [5]. Two samples collected from two infants after 1 and 3 months of monitoring also contained MDRKP (Table 1). All 8 neonates were discharged from hospital at 4-5 days of life in a satisfactory condition. Blood in stool and liquid stool were reported only once for the patient #1 on the third month of life, and constipation was reported for the patient #2 on the first month of life. No other manifested and prolonged symptoms were reported across the whole monitoring time from the individuals.

### 2.3. Phenotype Characterization

The antimicrobial susceptibilities of the 10 microbial isolates were determined using a broth microdilution procedure. The following antibacterial agents were tested: aminoglycosides (amikacin, netilmicin, gentamicin), *β*-lactam (amoxicillin-clavulanic acid, ampicillin, aztreonam, ceftriaxone, imipenem, meropenem), nitrofuran derivatives (nitrofurantoin), sulfonamides (sulfamethoxazole), 2, 4-diaminopyrimidines (trimethoprim), fluoroquinolones (ciprofloxacin), chloramphenicol, and fosfomycin. The production of extended-spectrum *β*-lactamases (ESBL) and susceptibility to *Klebsiella* phage and pyo bacteriophage were also analyzed during the routine. The results were interpreted in automated mode using a VITEK 2 Compact analyzer (bioMérieux SA, France) according to the producer's guidance documents.

### 2.4. Whole-Genome Sequencing and Assembly

Libraries were prepared using the NEBNext Ultra II DNA Library Preparation Kit. Whole-genome DNA was sequenced using the Illumina MiSeq platform (Illumina Inc., USA), with a paired-end run of 2 by 250 bp. Raw reads quality control was performed using FastQC v0.11 [6]; then, reads were trimmed using Trimmomatic v0.39 [7] and Cutadapt v2.4 [8], assembled using SPAdes v3.9.1 [9]. Assembly statistics were calculated using the reference *K. pneumoniae* genome with RefSeq ID NC_016845.1.

### 2.5. Further Genome Assembly Processing

The per-sample chromosome and plasmid assemblies were merged, filtered, and deduplicated using in-house scripts. The resulting assemblies were submitted to NCBI. The assemblies were annotated locally using Prokka v1.13.7 [10] and remotely with the NCBI Prokaryotic Genome Annotation Pipeline (PGAP) [11]. The *in silico* multilocus sequence type (MLST) results were computed using SRST2 v0.2 [12] and Kleborate [13]. The virulence-associated genes encoding yersiniabactin, aerobactin, salmochelin, colibactin, regulators of mucoid phenotypes, serotypes, and drug resistance determinants were combined using Kleborate with Kaptive subroutine [14]. Pangenome analysis was performed across the sequence query containing also 365 *K. pneumoniae* completed genome assemblies downloaded from the NCBI FTP server. A phylogeny was drawn using Roary, Pan Genome Pipeline v3.12.0 [15].

## 3. Results and Discussion

Neonatal infection is a clinical syndrome, characterized by systemic symptoms of the first month of life. However, in this study, we observed the case of newborn infant gastrointestinal tract colonization by the known pathogen *K. pneumoniae*, demonstrating a multidrug resistance phenotype without manifested symptoms.

The microdilution method has confirmed isolates with resistance to aminoglycosides, *β*-lactam, nitrofuran, fluoroquinolones, sulfonamides, trimethoprim, and fosfomycin antibiotics and *Klebsiella* phage. All the isolates were susceptible to amikacin, chloramphenicol, and pyo bacteriophage (Table 1).

The discovered WGS *de novo* resistome profile has included genetic determinants of drug resistance to aminoglycosides, fluoroquinolones, macrolides, sulfonamides, chloramphenicols, tetracyclines, and trimethoprim. It also confirmed the common *β*-lactamase (BLA) gene spread across all the isolates as well as the occurrence of isolates carrying certain genes of broad-spectrum *β*-lactamases (BSBL), BSBL with resistance to *β*-lactamase inhibitors (BSBL-inhR), and ESBL (Table 2). The results of the screening for genetic determinants of resistance to colistin, fosfomycin, glycopeptide, nitroimidazole, rifampicin, and a possible production of carbapenemases or ESBL with resistance to BLA inhibitors were negative.

**Figure 1:**
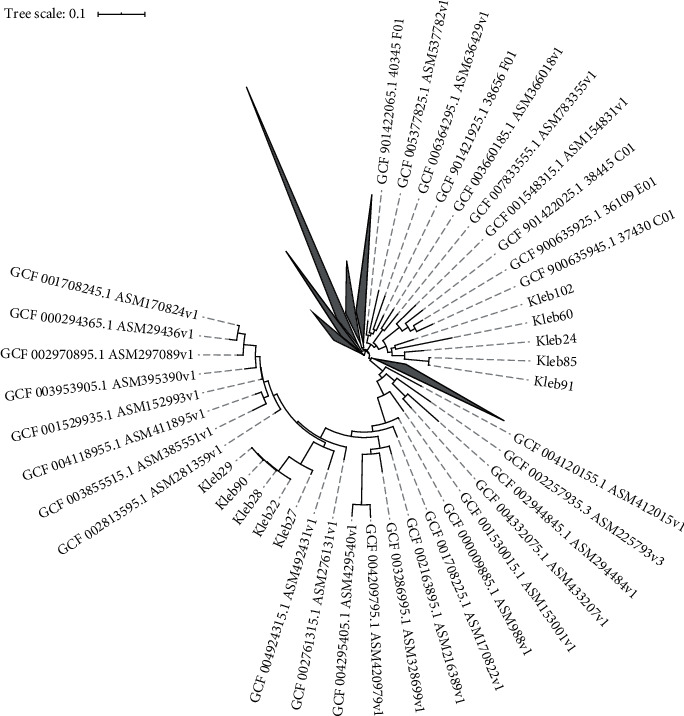
Phylogeny tree showing the relationship between 10 studying *K. pneumoniae* isolates and reference strains; built using Roary, the pangenome software; visualized using iTOL.

Biotype profiling has revealed that 10 isolates were related to 31 reference strains and combined into two clusters (Figure 1). The first cluster has included samples ##60, 85, 91, 102, and 24. All the isolates except the last one were obtained from cesarean delivery infants. The MLST analysis results have included the STs 37, 45, 268, and 9831LV (Table 2). The ST37 strains were reported as possible reservoirs for carbapenem resistance genes during antimicrobial therapy in neonates [16]; ST45, as the ESBL carrying infectious agents of highly contagious neonatal sepsis [17]; ST268, as possible reservoirs for New Delhi metallo-BLA (NDM) [18]; and ST983, as typical causative agents of nosocomial infections also harboring ESBL [19].

Due to the genetic divergence within the group, the patients most likely were infected from different sources. Because of the lack of the microbiological data from detailed examination performed on their mothers, we also cannot exclude the vertical transmission case after the silent colonization of the maternal urinary tract by MDRKP. It is known that asymptomatic bacteriuria occurs in 2–7 % of pregnant women in the United States and may cause severe urinary tract infection even leading to nephrectomy [20]. Gravidas are experiencing a 20-fold increased risk of pyelonephritis mainly caused by pregnancy immunosuppression, mechanical bladder compression, and ureteral dilatation [21]. The vertical transmission of pathogenic microorganisms is possible during the birth and before. The described cases of uterine *K. pneumoniae* infection during pregnancy include penetration via the fetal membranes and hematoplacental barrier, resulting to chorioamnionitis [22] and acute placental infection [23]. The infection source for the first group of studying infants remains unclear, due to the fact that their mothers were reported as healthy, with no symptoms or complains.

The other five isolates, ##22, 90, 27, 28, and 29, obtained from the infants born vaginally have formed even a less-divergent clade (Figure 1), allowing us to make a conclusion about a single MDRKP infection source. All 5 isolates were also positive for the genes of siderophore yersiniabactin (*ybt*), aerobactin (*iuc*) and salmochelin (*iro*), genotoxin colibactin (*clb*), hypermucoid determinants (*rmpA*, *rmpA2*), and specific *wzi* loci alleles related to the K- (capsule) and O- (LPS) antigen development, which cumulative presence corresponds to extremely virulent phenotypes in theory. More important, their MLST was only ST23. First reported in the mid-1980s in Taiwan, ST23 was often mentioned to present and strongly associated with the K1 capsular serotype [24]. The *K. pneumoniae* ST23 strains were confirmed as multidrug-resistant hypervirulent pathogens causing abscesses of the kidneys, pancreas, liver [25], and endogenous endophthalmitis [26], with dominance in the Asian Pacific region [27]. In fact, it means the 4 cases of asymptomatic colonization of the infant intestinal tract by hypervirulent strains of MDRKP that spread a short time ago and may be accounted as a hospital-acquired infection. The persistence of the MDRKP ST23 strain has been also confirmed for the patient #1 (sample #90) at the third month of life (Table 1)—still without acute immune response.

The fetal immune system is not developed compared with later life mostly due to the environmental limitation—the stress of the own remodeling tissues and noninherited maternal alloantigens should not provoke the immune reaction from the fetus side [28]. Their innate immune system is muted [29], the humoral immune responses are blunted, the immunoglobulin class switching is incomplete, and the released IgG antibodies decline rapidly after immunization [30]. However, the immature Th1-type T-cell response is compensated by the IFN-*γ* producing *γδ*-T cells [31]. By the way, the main mechanism of neonatal immune protection is based on vertical transport of antibodies via breastfeeding. With some fortune, breastfed newborns will not suffer infections that have induced the immune response from their mothers earlier. Otherwise, a lack of specific antibodies will result in infection development [32].

## 4. Conclusion

The emergence and spread of hypervirulent *K. pneumoniae*-carrying multidrug resistance genes in a hospital setting have constant dangerous context. We have demonstrated a phenomenon of silent carriage of two *K. pneumoniae* strain groups within the gut microbiome of healthy full-term neonates—more-divergent “normal” groups with multiple possible transmission paths and less-divergent hypervirulent strains with a single possible source. An undeveloped infant infection may indicate the successful production of the required antibodies by a maternal organism. It is possible after immunization of maternal organism occurred definitely before the childbirth—in maternity hospital settings or even before the hospitalization. Retrospectively, no infection source has been declared by hospital personnel after a time.

## Figures and Tables

**Table 1:** Patient data, sample data, and demonstrated drug resistance of the studied *K. pneumoniae* isolates.

Sample name	Kleb102	Kleb22	Kleb24	Kleb27	Kleb28	Kleb29	Kleb60	Kleb85	Kleb90	Kleb91
Sample number	102	22	24	27	28	29	60	85	90	91
Delivery	C	V	V	V	V	V	C	C	V	C
Patient ID	2	1	3	4	5	6	7	8	1	2
Patient age, days	33	3	3	3	4	3	4	4	90	3
*K. pneumoniae*, log_10_(CFU/*g*)	7	9	8	8	9	8	9	9	8	8
ESBL	-	+	-	-	+	+	+	+	-	+
Klebsiella phage	R	S	R	R	N	R	S	S	R	R
Pyo phage	S	S	S	S	S	S	S	S	S	S
Amikacin	S	S	S	S	S	S	S	S	S	S
Amoxicillin-clavulanic acid	R	R	S	S	R	R	R	R	S	R
Ampicillin	R	R	R	R	R	R	R	R	R	R
Aztreonam	R	R	S	S	R	R	R	R	N	R
Ceftriaxone	R	R	S	S	R	R	R	R	S	R
Chloramphenicol	N	S	S	S	S	S	S	S	S	S
Ciprofloxacin	S	I	S	S	R	I	S	S	S	S
Fosfomycin	R	R	I	R	R	R	R	R	N	R
Gentamicin	S	S	S	N	R	R	S	S	N	S
Imipenem	R	S	S	I	R	I	R	R	N	R
Meropenem	R	S	S	S	S	S	S	S	N	S
Netilmicin	S	R	S	S	R	R	S	S	N	I
Nitrofurantoin	R	I	S	R	R	I	R	R	R	R
Sulfamethoxazole	S	R	S	S	R	R	R	R	N	R
Trimethoprim	S	R	S	S	R	R	R	R	N	R

CFU, colony-forming unit. Delivery: V, vaginal; C, cesarean section. Susceptibility to an antimicrobial agent: S, susceptible, R, resistant; I, intermediate; N, not defined.

**Table 2:** Genome assembly data, MLSTs, and drug resistance determinants of the studied *K. pneumonia* isolates.

Sample name	Kleb102	Kleb22	Kleb27	Kleb24	Kleb28	Kleb29	Kleb60	Kleb85	Kleb90	Kleb91
GenBank Accession ID	VOOJ00000000	VOOA00000000	VOOD00000000	VOOC00000000	VOOE00000000	VOOF00000000	VOOG00000000	VOOH00000000	VOOB00000000	VOOI00000000
SRA accession ID	SRR12102917	SRR12102926	SRR12102923	SRR12102924	SRR12102922	SRR12102921	SRR12102920	SRR12102919	SRR12102925	SRR12102918
Reads number	1040088	805318	1056762	856300	926382	1259976	994296	1207354	1216382	1381726
Coverage	58.7x	45.4x	59.6x	48.3x	52.3x	71.1x	56.1x	68.1x	68.6x	78.0x
Genome assembly length	5297684	5881113	5641111	5518356	5885411	5890967	5666743	5324705	5882697	5326604
Contig number	106	111	76	94	120	118	91	74	108	77
N50 metric	350001	307112	322041	319410	307112	307112	263911	358425	307112	437151
Largest contig length	1106296	770102	1334922	930837	771547	799268	642551	812552	799226	812552
MLST	ST983-1LV	ST23	ST23	ST37	ST23	ST23	ST268	ST45	ST23	ST45
Yersiniabactin	ybt unknown	ybt 1; ICEKp10	ybt 1; ICEKp10	—	ybt 1; ICEKp10	ybt 1; ICEKp10	ybt 17; ICEKp10	—	ybt 1; ICEKp10	—
Colibactin	—	clb 2	clb 2	—	clb 2	clb 2	clb 3	—	clb 2	—
Aerobactin	—	iuc 1	iuc 1	—	iuc 1	iuc 1	iuc 1	—	iuc 1	—
Salmochelin	—	iro 1	iro 1	—	iro 1	iro 1	iro 1	—	iro 1	—
*rmpA*	—	rmpA_2 (KpVP-1), rmpA_2 (KpVP-1)	rmpA_2 (KpVP-1)	—	rmpA_2 (KpVP-1)	rmpA_2 (KpVP-1)	rmpA_2^*∗*^(KpVP-1), rmpA_2^*∗*^(KpVP-1)	—	rmpA_2 (KpVP-1)	—
*rmpA2*	—	rmpA2_8, rmpA2_8	rmpA2_5, rmpA2_5	—	rmpA2_8, rmpA2_8	rmpA2_8, rmpA2_8	rmpA2_2^*∗*^, rmpA2_2^*∗*^	—	rmpA2_8, rmpA2_8	—
*Wzi*	wzi39	wzi1	wzi1	wzi123	wzi1	wzi1	wzi95	wzi101	wzi1	wzi101
K Locus	KL39	KL1	KL1	KL136	KL1	KL1	KL20	KL24	KL1	KL24
O Locus	O3b	O1v2	O1v2	O2v2	O1v2	O1v2	O2v1	O2v1	O1v2	O2v1
Aminoglycosides	StrB; StrA^*∗*^	StrB; StrA^*∗*^; Aac3-IIa^*∗*^; StrB; StrA^*∗*^	—	StrB; StrA	StrB; StrA^*∗*^; Aac3-IIa^*∗*^	StrB; StrA^*∗*^; Aac3-IIa^*∗*^	StrB; StrA^*∗*^; Aph3-Ia^*∗*^	StrB; StrA^*∗*^	StrB; StrA^*∗*^; Aac3-IIa^*∗*^; StrB; StrA^*∗*^	StrB; StrA^*∗*^
Fluoroquinolones	—	QnrB1?	—	—	QnrB1?	QnrB1?	—	—	QnrB1?	—
Macrolides	—	—	—	—	—	—	EreA2	—	—	—
Phenicols	CatA1^*∗*^	CatB4	—	—	CatB4	CatB4	—	CatB4	CatB4	CatB4
Sulfonamides	SulII^*∗*^	SulII; SulII	—	—	SulII	SulII	SulI; SulII^*∗*^	SulII	SulII; SulII	SulII
Tetracyclines	TetA	TetA	—	—	TetA	TetA	—	—	TetA	—
Trimethoprim	—	DfrA14; DfrA14	—	—	DfrA14	DfrA14	DfrA5	DfrA14	DfrA14	DfrA14
BLA	SHV-187^*∗*^; AmpH^*∗*^	SHV-190^*∗*^; AmpH; OXA-1	SHV-190^*∗*^; AmpH	AmpH^*∗*^	SHV-190^*∗*^; AmpH; OXA-1	SHV-190^*∗*^; AmpH; OXA-1	AmpH^*∗*^	AmpH^*∗*^; OXA-1	SHV-190^*∗*^; AmpH; OXA-1	AmpH^*∗*^; OXA-1
ESBL	—	CTX-M-15; CTX-M-15	—	—	CTX-M-15	CTX-M-15	SHV-13^*∗*^; CTX-M-3; CTX-M-3	CTX-M-15	CTX-M-15; CTX-M-15	CTX-M-15
BSBL	—	—	—	SHV-77^*∗*^	—	—	—	—	—	—
BSBL-inhR	TEM-30^*∗*^	TEM-30^*∗*^; TEM-30^*∗*^	—	—	TEM-30^*∗*^	TEM-30^*∗*^	—	SHV-26^*∗*^; TEM-30^*∗*^	TEM-30^*∗*^; TEM-30^*∗*^	SHV-26^*∗*^; TEM-30^*∗*^

MLST, in silico multilocus sequence type; BLA, *β*-lactamases; BSBL, broad-spectrum *β*-lactamases, BSBL-inhR, BSBL with resistance to *β*-lactamase inhibitors, ESBL, extended-spectrum *β*-lactamases. Asterisked proteins are ones without exact nucleotide and amino acid match.

## Data Availability

The corresponding WGS project has been deposited at the NCBI GenBank database under the main BioProject accession ID PRJNA556398. The project data analysis scripts and materials are hosted on GitHub (https://github.com/ivasilyev/curated_projects/tree/master/inicolaeva/klebsiella_infants).
